# The Mineral Profile of Polish Beers by Fast Sequential Multielement HR CS FAAS Analysis and Its Correlation with Total Phenolic Content and Antioxidant Activity by Chemometric Methods

**DOI:** 10.3390/molecules25153402

**Published:** 2020-07-27

**Authors:** Elżbieta Zambrzycka-Szelewa, Edyta Nalewajko-Sieliwoniuk, Mariusz Zaremba, Andrzej Bajguz, Beata Godlewska-Żyłkiewicz

**Affiliations:** 1 Faculty of Chemistry, University of Bialystok, Ciołkowskiego 1K, 15-245 Białystok, Poland; elazamb@uwb.edu.pl (E.Z.-S.); zaremba_mariusz@onet.pl (M.Z.); 2 Faculty of Biology, University of Bialystok, Ciołkowskiego 1J, 15-245 Białystok, Poland; abajguz@uwb.edu.pl

**Keywords:** sequential multielement determination, phenolic compounds, antioxidant activity, beer, chemometric analysis

## Abstract

Beer is the most common alcoholic beverage worldwide, and is an excellent source of macro- and microelements, as well as phenolic compounds. In this study, a fast method for the determination of Na, K, Mg, Ca, Fe, Mn, and Cu in beer was developed using flame atomic absorption spectrometry. The precision of this method was between 0.8 and 8.0% (as the relative standard deviation (RSD)), and limits of detections were in the range of 0.45 (Mn)–94 µg/L (Na). Among the macroelements tested in the beer samples, K was found at the highest concentration, whereas Na was found at the lowest concentration level. Beer also turned out to be a good source of Mg and K. The total phenolic content (TPC) was determined by the Folin–Ciocalteu method, while the antioxidant activity was estimated by the ABTS method. The results show remarkable variations in the mineral content, TPC, and antioxidant activity across the beer types and brands. Moreover, the relations between the type, color, refraction index, antioxidant activity, extract, alcohol, mineral, and the total phenolic contents were investigated using the factor analysis of mixed data (FAMD) combined with hierarchical clustering on principal components (HCPC).

## 1. Introduction

Beer is one of the most popular alcoholic beverages in the world. The consumption of beer in Poland totals an average of 97 L per person and solidifies Poland’s place as the fourth-highest consumption country in the European Union [1]. Compared to other alcoholic beverages, beer has a higher nutritional value because it contains many macro- and microelements, as well as some vitamins, carbohydrates, gluten, and antioxidants [2]. Metals in beer can originate from the brewing water, malt grains, hops, fruits, and spices. Some salts (e.g., CaSO_4_, MgSO_4_, ZnSO_4_, and CaCl_2_) are added throughout the brewing process in order to control pH, adjust the taste, improve efficiency, and enhance the fermentation performance. Sources of metals in beer have been studied, and several investigators have examined their fate during the brewing, fermentation, and clarification processes [3,4]. It is known that numerous metals play an important role in the physiological processes of yeast. Metals like Zn, Fe, and Cu are cofactors in over 100 enzymatic reactions; they govern protein synthesis and the phospholipid composition of membranes in yeasts. They are important for yeast growth and metabolism, as they influence the fermentation rate and stabilize enzyme, protein, and membrane systems. Moreover, certain elements can affect the quality and taste of the final beer products [2]. It is known that high Fe content reduces yeast activity and causes the metallic taste of beer, whereas dissolved Cu reduces sulfur flavors and aromas in beer due to sulfide reactions. Moreover, the oxidizing properties of Fe, Cu, and Mn may decrease the shelf-life of the final beer [5]. Some metals, especially Pb and Cd, may be harmful above a certain concentration. The total allowed content of metals in brewing liquors and beer is regulated, so the analysis of metals is essential.

Beer is a complex mixture of compounds with antioxidant activity, originating mainly from raw materials, but also formed during the brewing process [6]. Natural antioxidants present in raw materials include phenolic compounds, Maillard reaction products (reductones and melanoidins), thiols, sulfites, sugars, carotenoids, vitamins, and chelating agents. Phenolic compounds, which are products of the secondary metabolism of plants, are responsible for 55.0–88.1% of the total antioxidant capacity of beer. They display many health benefits and protect our bodies against cardiovascular diseases, certain types of cancers, and aging-related disorders [7]. Different types and brands of beers have similar phenolic profiles. However, significant variations between the total and individual phenolic contents are most likely due to a difference in the variety and quality of raw materials, as well as dissimilar malting and brewing processes. Beer is generally considered one of the major sources of phenolic compounds; its total content usually exceeds 100 mg gallic acid equivalents (GAE) per liter. Around 80% of the phenolic compounds originate from barley malt, and the remaining 20% are derived from hops. Phenolic compounds have an influence on beer color, foam, and colloidal and sensory properties. Moreover, through metal chelation and free radical scavenging, phenolic compounds protect beer against oxidative staling and improve its physical stability and flavor. Phenolics in beer include several classes of compounds, such as phenolic acids, flavanols, proanthocyanidins, flavones, prenylchalcones, flavanones, alpha-acids, and stilbenes.

The most popular method for determining the total phenolic content (TPC) in beer is the Folin–Ciocalteu (FC) assay, which is based on the reduction of the FC reagent (a mixture of phosphomolybdate and phosphotungstate) by phenolics and the measurement of the absorbance at 725–760 nm [8]. The FC reagent does not only react with phenolic compounds, but also reacts with other reducing substances (amino acids, aldehydes, ketones, amines, nucleotides, proteins, thiols, carbohydrates, and vitamins), therefore, the assay enables one to measure the overall reducing capacity of beers. However, because of the fact that phenolic compounds are the most abundant antioxidants in beers, FC assays give a rough approximation of the total phenolic content. For proper evaluation of the total antioxidant activity of beers, spectrometric analytical methods, such as 2,2′-azinobis(3-ethylbenzothiazoline-6-sulfonic acid) diammonium salt radical cation scavenging activity (ABTS) assay, 1,1-diphenyl-2-picrylhydrazyl (DPPH) radical scavenging activity assay, oxygen radical absorbing capacity (ORAC) assay, and ferric reducing antioxidant power (FRAP) assay are used due to their simplicity, sensitivity, and short time of analysis.

Typical techniques used for the determination of metals in beers include flame atomic absorption spectrometry (FAAS) [9,10,11], graphite furnace atomic absorption spectrometry (GFAAS) [12,13], inductively coupled plasma optical emission spectrometry (ICP-OES) [9,10,14,15], and inductively coupled plasma mass spectrometry (ICP-MS) [3,16,17,18]. Since direct aspiration of beer results in flame/plasma fluctuations, the formation of solid deposits on the burner head [9,12], or clogging of the nebulizer, the analysis of metals is usually performed in digested samples. Digestion of beer was carried out using concentrated HNO_3_ [16,17], or mixtures of HNO_3_ and 30% H_2_O_2_ [9,11,15,19], H_2_SO_4_ and H_2_O_2_ [10], and HF [3] in open and closed systems. In order to minimize the influence of organic residue on the efficiency of atom/ion formation of elements, the standard addition method is usually recommended for calibration.

The typical sensitivities of FAAS do not allow the adoption of this strategy when determining major and trace elements. Other drawbacks of the classical line-source FAAS technique are the single-element character and narrow range of calibration curves. The development of high-resolution continuum source flame atomic absorption spectrometry (HR CS FAAS) allowed for sequential multielement analyses that are particularly important for routine laboratories dedicated to large-scale food control because of time and analytical cost reduction. Using this technique, it is also possible to determine each analyte under optimized conditions in a single run, as the flame composition, stoichiometry, and burner height can be changed very fast. This approach is clearly preferable to the simultaneous determination of elements under compromised conditions. Moreover, the intensity of the Xe lamp (continuous source) is practically the same in the whole spectrum; thus, less sensitive lines can be used for the determination of elements. This is beneficial as the sensitivity might be accommodated in such a way that all the elements of interest can be monitored sequentially without the need for diluting the samples. Furthermore, it is also possible to use side pixels to decrease sensitivity [20,21,22]. For all these reasons, HR CS FAAS is considered an appropriate tool for performing the fast multielement determination of metals in various samples. Although several methods have been developed for sequential determination of elements in soil extracts [21], digested soils [23], digested plant leaves [24,25], seawater [26], or apple juices [27], the HR CS FAAS technique has not been applied to the analysis of beer samples yet.

A literature review reveals that there are studies that describe different correlations occurring in beer. The influence of the beer type and place of origin on the alcohol and metal contents was investigated by Alcázar et al. [28] and Rodrigo et al. [18]. Wyrzykowska et al. [16] studied the interdependencies among trace metals in beers. Alcázar et al. [9] examined the correlations between the mineral content and the type of beer (lager, dark, and low alcoholic). Moura-Nunes et al. [29] investigated the impact of the type and style of beer, ethanol content, refractive index, and bitterness on the content of phenolic compounds. While Polak et al. [30] studied the influence of the color, the type, and the content of the extract and alcohol on the antioxidant activity of beers. Nevertheless, there is only one paper describing the mineral and the total phenolic contents in beer samples [31]. However, the correlations between these analytes were not investigated. 

The objectives of this study were:

- To develop the fast FAAS method for sequential determination of macro- and microelements in beers, which could be used in routine laboratories for food analysis,
- To study the total phenolic content and antioxidant properties of Polish beers in relation to the type, color, alcohol and extract contents, and method of fermentation,
- To investigate the possible relationship between the macro- and microelements and phenolic compounds.

For the purpose of this study, 29 beers produced by leading Polish brewery companies and small local breweries were analyzed. The relations between the type, color, refractive index, antioxidant activity, extract, alcohol, mineral, and total phenolic contents were investigated using the factor analysis of mixed data (FAMD) combined with hierarchical clustering on principal components (HCPC).

## 2. Results and Discussion

### 2.1. Development of Sequential Methods for the Determination of Macro- and Microelements in Beer

HR CS FAAS can be considered a suitable tool for performing fast multielement analyses in various matrices. However, determination of macro- and microelements in beer in a single run may be difficult as their concentrations differ by 2–3 orders of magnitude [2,32]. In order to avoid these problems, the measurement conditions were optimized for spectral lines of different sensitivities, i.e., main spectral lines (100% intensity) of Zn, Fe, Cu, Mn, Ca, and secondary spectral lines of Na (0.48% intensity), K (0.5% intensity), and Mg (4.3% intensity). Initially, the flame composition, ratio of air-acetylene flow rates, and burner height were adjusted for each element individually in order to obtain the highest sensitivity of measurements. Sequential analysis of elements was performed in the following order: Cu, Zn, Fe, Mg, Na, K, Mn, and Ca using optimal measurement conditions (listed in Section 2.4). Since the analytical line of Zn overlaps with the molecular absorption band of NO if the air-acetylene flame is used for atomization [24], all elemental standards were prepared in 1% HCl. The influence of wavelength integrated absorbance on the sensitivity of element determination was studied by varying the number of pixels from 1 to 11, i.e., from CP (central pixel) to CP ± 9. The optimal number of measurement pixels was selected based on the background signal, repeatability of measurements, and limit of detection of each analyte. The characteristic parameters of the method, such as the linear range of the calibration graphs, working calibration range, precision, limit of detection, and limit of quantification were also estimated for each element. For evaluation of calibration graph linearity, multielemental standards in the concentration range of 0.01–20 mg/L (for microelements) or 1–200 mg/L (for macroelements) were prepared. Typical linear correlation coefficients of calibration graphs were higher than 0.998. The limit of detection (LOD) was defined as 3SDblank/a, where SD is the standard deviation of the blank measures, and a is the slope of the calibration graph. The limit of quantification (LOQ) was calculated as 10SDblank/a. The within-day precision of absorbance measurements, expressed as the relative standard deviation (RSD) for six independent measurements of the standard solution of the target element, was below 3.2%. The between-day precision, calculated as the RSD (in %) of calibration graph slopes recorded in 5 different days, was below 8%. The main figures of merit of sequential determination of eight elements by HR CS FAAS are shown in Table 1. This method is sensitive and very fast, as the determination of eight elements in one sample takes under 7 min.

In order to decrease the sensitivity of Mg determination and concurrently increase the linearity of the calibration graph used for the sequential multielement determination in beer, a two side pixel registration approach was tested. Typically, for the best sensitivity, the central pixel approach is preferred. When higher masses of analyte are introduced into the flame, the use of side pixels may become advantageous [21,24]. Therefore, the mathematical subtraction of absorbance of Mg registered for CP ± 1 and CP was carried out (i.e., absorbance was measured using the sum of the detector pixels 100 and 102). This operation is possible due to the fact that the software is capable of storing reference spectra and of subtracting spectra from these recorded for the sample. As a result, the sensitivity of Mg measurements decreased by about 60%, whereas the linearity of the calibration graph was extended from 7 to 12 mg/L. Such an approach allows for the determination of higher analyte concentrations due to an extension of the working range of HR CS FAAS. The parameters obtained for the determination of Mg at 202.582 nm using a different number of pixels are presented in Table S1 from Supplementary Material. The limit of detection and precision of the method based on the registration of two side pixels (LOD equaled 77 µg/L) was worse than that obtained for methods based on the registration of three or five pixels (e.g., 58 µg/L and 60 µg/L, respectively) due to the increased noise that was added to the total integrated absorbance. However, this methodology allowed for sequential determination of three macroelements: K, Na, and Mg in beer samples. The reproducibility of results was below 2.8%.

The effect of the beer matrix on the determination of elements using the developed method was examined for two different beer samples (13 and 19) containing medium concentrations of extract, alcohol, and sugar (see Table 2). The concentration of elements in each beer (10-fold diluted in 1% HCl) was determined using two calibration techniques: the calibration graph and the standard addition method (using four standard additions). Slopes of both calibration functions were the same in the range of analytical error for K, Na, and Mg. The concentration of elements in the beers that were obtained by two calibration methods varied less than 4% (0.7% for Mg, 2% for K, and 4% for Na). Therefore, the external calibration graph method might be recommended for sequential determination of K, Na, and Mg by HR CS FAAS in beer samples diluted with 1% HCl or HNO_3_. The repeatability of results, calculated as RSD (in %) of the concentration of analyte determined in three independent beer samples, was below 2%.

The strong interference of the beer matrix on the atomization of Ca was eliminated by the addition of 1% La as a releasing agent [25]. In this way, the slope of the calibration graph for Ca decreased by 30% (Table 1), but the results obtained by two calibration methods were consistent (within 5%).

Using the two calibration methods, the concentration of microelements in beers varied approximately 10% for Fe, 12% for Mn, 34% for Cu, and 44% for Zn. A similar effect of the beer matrix on analytical signals of Mn, Fe, and Cu was observed by Bellido-Milla et al. [10]. This effect was minimized through the digestion of samples with nitric acid. Therefore, in order to overcome the matrix effect and avoid using the standard addition method, beer samples were digested in a conventional open vessel with nitric acid and hydrogen peroxide on a hot plate [11,33]. The final product was a clear solution in a pale yellow color. Concentrations of Fe, Cu, and Mn in such digested beer samples determined by HR CS FAAS using both calibration procedures were in good agreement (differences below 5%). However, due to a serious interference of nitric acid on the determination of Zn, this element was excluded from further studies. The analytical characteristic of a sequential method for determination of Fe, Cu, and Mn in 10% nitric acid is presented in Table 1.

Due to a lack of reference material of beer with certified concentrations of elements, the trueness of developed methods was verified by the determination of target elements in the certified reference material of mixed Polish herbs (MPH-2). The MPH-2 material was selected since it contains similar concentrations of elements to beer samples. The results presented in Table S2 from Supplementary Material demonstrated good accuracy of developed methods. Recoveries of macroelements were in the range of 97.4–100.6%, while microelements were in the range of 91.6–103.7%. The repeatability of the determination of elements expressed as the relative standard deviation of three independent analyses of the same beer sample was in the range of 1.1–7.3%.

In summary, due to the serious interference of the beer matrix on the analytical results, two methods were developed for sequential determination of macro- and microelements in beer. For sequential determination of Na, K, and Mg, beer samples were simply diluted with 1% HCl, while before sequential determination of Fe, Mn, and Cu, beer samples were submitted to a wet mineralization procedure. The external calibration procedure based on standard solutions prepared in 1% HCl (for macroelements) or 10% HNO_3_ (for microelements) was applied for quantification of investigated elements in beer samples. The addition of a La modifier is crucial for accurate determination of Ca in beer. The analytical parameters (in terms of sensitivity, precision, and LOD) of developed methods for sequential determination of macro- and microelements are similar or better in comparison to those obtained in other papers [11,21,24].

### 2.2. Determination of the Total Content of Macro- and Microelements in Beer

The total content of macro- and microelements in investigated beer samples was determined according to the procedures described in Section 3.4.1 and Section 3.4.2. The samples were analyzed in triplicate. The results of HR CS FAAS assays are presented in Table 2. Based on the obtained results, it was concluded that K was found in the beer samples at the highest concentration level among the macroelements. Its concentration in beer was in the range from 367 ± 10 mg/L (beer 16) to 855 ± 16 mg/L (beer 27). On the basis of the results obtained in this study, it could be concluded that the analyzed beers may serve as a significant source of K for humans. According to the dietary reference intakes recommended by US Food and Drug Administration [34] the intake levels for K should be about 3500 mg/day for adults. This means that 500 mL of beer (one bottle) covers from 5.3 to 12% of the daily US norms. Similar studies carried out by Rajkowska et al. [35] on Polish beers also revealed high concentrations of K (from 172 to 518 mg/L) in comparison to beers from countries such as Portugal, Thailand, Italy, Vietnam, Romania, Spain, and Germany [17,28,36], but overall lower than the values obtained in this study. Higher content of K was only found in beers from Britain (from 135 to 1100 mg/L) [17]. The level of K may vary depending on a different class of yeast used during the production process (which are a significant source of K), as well as a different quality of resources [16]. The level of K has to be strictly controlled, because a potassium concentration higher than 500 mg/L may inhibit the activity of enzymes, causing a salty taste of beer and thus reducing its quality [36]. On the contrary, the lowest contents among the macroelements were found for sodium. The concentration of this metal ranged from 7.75 ± 0.04 mg/L (beer 20) to 74.2 ± 1.3 mg/L (beer 4). A low sodium concentration in beer affects the sweetness and smoothness of its taste. According to the US standards, the intake of sodium for humans should be about 2.4 g a day [34]. Therefore, one bottle of beer produced in Poland may cover up to 1.5% of the daily need for this element. The concentration of calcium in the tested beer samples ranged from 19.1 ± 1.6 (beer 20) to 117 ± 4 mg/L (beer 23). Comparing these values to the norms of recommended calcium intake, which are 1000 mg per day [34], it turns out that one 500 mL bottle of beer may cover up to 5.9% of the daily requirement in terms of this element. Calcium had no significant effect on the taste of beer. The concentration of magnesium was in the range from 64.0 ± 1.0 (beer 2) to 169 ± 0 mg/L (beer 26). Such Mg concentration values can cause a bitter taste in beer. However, taste also depends on the ratio of calcium and magnesium concentration in this beverage [37]. While comparing the obtained results with the daily needs, it was concluded that a single bottle of beer covers between 8.0% and 21.1% of the daily requirement of magnesium, which is 400 mg/day for adults [34]. Comparing the results obtained in this study with the values found in the literature (Table 3), it can be concluded that all macroelement concentrations were within the ranges obtained for beers from other countries. Only in the case of K its concentration was almost the same for each kind of beer. While bottom-fermented beers contained higher concentrations of Mg (125 ± 22 mg/L) and Ca (73.9 ± 28.9 mg/L) when compared to top-fermented beers (Mg: 103 ± 20 mg/L, Ca: 41.6 ± 12.7 mg/L, Na: 42.2 ± 18.4 mg/L), they also contained lower concentrations of Na (31.5 ± 19.0 mg/L). A similar situation was observed for dark beers, especially porters and bocks, which also contained higher concentrations of Mg (143 ± 24 mg/L (porters), 130 ± 9 mg/L (bocks)) and Ca (91.7 ± 20.7 mg/L (porters), 86.9 ± 33.9 mg L^−1^ (bocks)) and lower concentrations of Na (25.5 ± 9.2 mg/L (porters), 19.0 ± 10.6 mg/L (bocks)) compared to pale beers (Mg: 110.5 ± 24.4 mg/L, Ca: 52.7 ± 23.9 mg/L, Na: 31.7 ± 18.2 mg/L). Rodrigo et al. [18] found that lagers (bottom-fermented beers) had the lowest content of Ca, K, Mg, and dark beers (stout/porter), as well as the highest content of Mg. While the beer style did not have a significant effect on the concentration of Na.

Among the microelements tested, Fe was present in investigated beer samples at the highest concentration levels, ranging from 62.4 ± 1.7 to 1872 ± 2 µg/L. Taking into account the US recommendations, the intake of Fe should be about 18 mg/day for adults [34]. Therefore, one bottle of beer may cover up to 5.2% of the daily need for this element. However, high Fe concentration in beer has a negative impact on its quality. A concentration above 0.3 mg/L causes a grey color of beer foam [2]. The metallic taste of beer becomes noticeable when the concentration of Fe exceeds 0.5 mg/L. This threshold value was exceeded by 3 of the tested beers (beers 5, 18, and 20). A concentration higher than 1 mg/L (beers 5 and 20) caused beer haze and accelerated the oxidation of organic compounds, leading to flavor changes of this beverage. Mn is the second microelement with the highest concentrations found in the tested beer samples. The concentration of this metal was in the range of 65.4 ± 0.2 (beer 17)–277 ± 1 µg/L (beer 16). In small concentrations (<0.1 mg/L), Mn is necessary for the proper growth of yeast involved in the beer fermentation process [18]. In process water, the manganese concentration should not exceed 0.2 mg/L, because it may negatively affect the colloidal stability of beer [35]. On the basis of the results obtained in this study, it can be concluded that a single bottle of beer may cover up to 6.9% of the daily need for Mn, which is about 2 mg/day [34]. The content of Cu in beer samples was in the range of 12.1 ± 0.1 (beer 28)–85.8 ± 3.1 µg/L (beer 9). Similar to Fe, high concentrations of Cu accelerated the oxidation of organic compounds present in beer. The US recommended adequate intake levels for Cu are about 2 mg a day for adults [34]. This means that 500 mL of beer may cover up to 2.1% of the daily norm in the USA. Comparing the results obtained in this work with the values found for Polish beers in the literature (Table 3), it can be concluded that for Fe and Mn, the values determined in this study were higher than those obtained by Wyrzykowska et al. [16], Pohl et al. [11], and Rajkowska et al. [35]. In contrast, the Cu levels were lower in this study than they were in the aforementioned works. However, the levels of investigated microelements were within the ranges obtained for beers brewed in other countries (Table 3). Among the microelements tested, the type of fermentation did not have an influence on the content of Mn and Cu. Only in the case of Fe was there a difference between the two types of fermentation processes. Top-fermented beers contained about two times higher concentration of this metal (431 ± 523 µg/L) compared to bottom-fermented beers (215 ± 108 µg/L). Dark beers exhibited a slightly higher content of Mn (168 ± 67 µg/L) but a lower content of Fe (302 ± 283 µg/L) and Cu (36.2 ± 18.0 µg/L). Especially low concentrations of these metals were found in bocks (Fe: 204 ± 137 µg/L, Cu: 41.3 ± 10.2 µg/L) and porters (Fe: 204 ± 137 µg/L, Cu: 41.3 ± 10.2 µg/L).

The results showed remarkable variations in mineral content between different types and brands of beers, with differences varying from a factor of 2 (K) to a factor of 30 (Fe). Concentrations of macro- and microelements in beer mainly depend on the quality of natural resources used in the brewing process (cereal, hops, yeast, water, and soil), application of different methods to process the raw material (type of fermentation), as well as the presence of environmental contaminants [16]. Taking into account the fact that several tested beer samples had high concentrations of iron and manganese, which had a negative impact on beer quality, a systematic monitoring of the content of these metals should be performed during the technological process of beer production.

### 2.3. Determination of the Total Phenolic Content and the Total Antioxidant Activity

The total content of phenolic compounds in beer samples was determined by the FC method (described in Section 3.4.3), while the antioxidant activity was estimated by the ABTS method (described in Section 3.4.4). The samples were analyzed in triplicate. The results of the spectrophotometric assays are given in Table 2. The TPC of the beer samples was in the range of 307 ± 16 (beer 22)–1266 ± 24 mg/L (beer 27). The average concentrations of TPC in pale and dark beers were 648 ± 127 and 845 ± 269 mg/L, respectively. The TPC was significantly higher in dark beers (Table 2 and Table 4). The highest values were measured in porter (927–1108 mg/L) and bock (894–1185 mg/L) beers. Our findings are in agreement with those of Dabina-Bicka et al. [38], Mitić et al. [7], and Piazzon et al. [39], who also observed a higher TPC in dark beers. The average concentrations of TPC in ale pale and dark beers were 687 ± 140 and 582 ± 192 mg/L, respectively. While the average concentrations of TPC in lager pale and dark beers were 630 ± 103 and 991 ± 178 mg/L, respectively. Neto et al. [40] and Vinson et al. [41] have found that ale group beers showed higher TPC and antioxidant activity compared to lager group beers. Pérez-Ráfols et al. [42] found that in general, ale beers were 15% richer in overall phenolic compounds. However, the authors did not specify what type of beers (pale, dark) was investigated. Contrary to the results obtained by other researchers [43], craft beers from small breweries did not contain a higher content of phenolic compounds. The results obtained by a FC assay were similar to those obtained for Polish beers by Pieszko et al. [44] (222–1185 mg/L) but higher than those obtained by Ditrych et al. [45] (115–408 mg/L), most likely due to the use of different methods for TPC determination. There are several studies on the content of phenolic compounds in beers from different countries. Dabina-Bicka et al. [38] found that in Latvian beers, values of TPC ranged from 301 to 864 mg/L, while Bertuzzi et al. [43] estimated TPC in Italian beers were within the range of 205–841 mg/L. Moura-Nunes et al. [29] found that Brazilian beer TPC was in the range of 164–572 mg/L, whereas Zhao et al. [46] reported that Chinese beers contained TPC in the range of 152–339 mg/L. In addition, Mitić et al. [7] found that Serbian beer TPC values were from 328 to 545 mg/L, whilst Pai et al. [47] reported TPC values of 160–620 mg/L in Indian beers. Nino-Medina et al. [48] found that Mexican beers contained TPC in the range of 174–274 mg/L. The content of phenolic compounds may vary depending on the quality and quantity of raw materials, the malting and brewing processing parameters, and the TPC evaluation methods used in the studies [44,49].

The antioxidant activity of investigated beer samples, determined by the ABTS method, also exhibited considerable differences ranging from 46.1 ± 3.1 (beer 2) to 146 ± 2 μmol/L (beer 23). The ABTS^•+^ scavenging activity of pale beers was in the range of 46.1 ± 3.1–104 ± 2 μmol/L. Dark beers revealed higher antioxidant capacity, ranging from 55.4 ± 0.5 to 146 ± 2 μmol/L, compared to light beers (Table 2 and Table 4). The highest values were obtained for bock (69.9–146 μmol/L) and porter (106–145 μmol/L) beers. Our findings are in agreement with those described in the literature [6,30]. Higher antioxidant properties of dark beers might be related to the use of dark malt, which contains heat-induced substances such as melanoidins and reductones that are formed by the Maillard reaction during kilning and roasting processes [6]. Similarly to TPC, the average antioxidant capacity of pale ale beers was higher (73.9 ± 21.0 μmol/L) than that of pale lager beers (64.8 ± 12.5 μmol/L). In the case of dark beers, the average antioxidant capacity of ale type beers was lower than that of lager type beers (Table 2). However, Polak et al. [30] stated that the kind of fermentation (bottom in ale, top in lager type beers) did not affect the antioxidant activity of beers. The obtained values of antioxidant activity were lower than those reported in the literature by Polak et al. [30] in Polish beers (711–3328 μmol/L), Mitić et al. [7] in Serbian beers (140–350 μmol/L), Nino-Medina et al. [48] in Mexican beers (140–230 μmol/L), Moura-Nunes et al. [29] in Brazilian beers (400–3020 μmol/L), Bertuzzi et al. [43] in Italian beers (300–600 μmol/L), and Zhao et al. [46] in Chinese beers (550–1950 μmol/L). The differences can be related to higher amounts of natural antioxidants present in raw materials (including phenolic compounds, carotenoids, thiols, and vitamins), the brewing process itself, or different procedures used for the determination of the total antioxidant capacity of beer samples.

The highest values of TPC and antioxidant activity were found in dark beers, especially porters and bocks. Therefore, dark beers, consumed in a limited amount, could be a rich source of phenolic antioxidants in our diet. The results showed remarkable variations in TPC as well as antioxidant activity across beer brands. The results of TPC and ABTS assays differed by factors of 4 and 3, respectively, which is similar to the variation registered by Zhao et al. [6,46]. In studies by Ditrych et al. [45], the differences in antioxidant activity of beers varied even by up to a factor of 12, which shows that producers may considerably improve the antioxidant properties of their products and improve their bioactivity through an appropriate selection of raw materials and changes in the brewing process.

### 2.4. Chemometric Analysis

**Figure 1:**
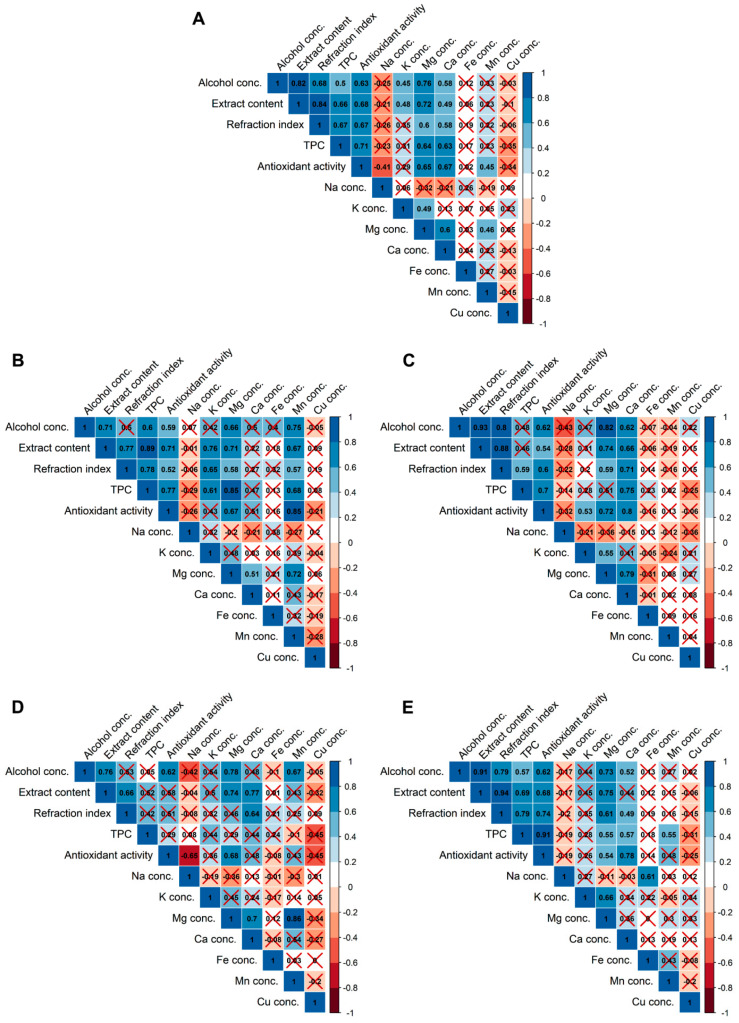
Correlation heatmap for unfiltered beer parameters (**A**; *n* = 29), pale (**B**; *n* = 15), dark (**C**; *n* = 14), top-fermented (**D**; *n* = 12), and bottom-fermented (**E**; *n* = 17) beers. The colored and labeled scale codes are for the value of the Spearman’s rank correlation coefficient r_s_. Positive correlations are blue, while negative correlations are red. However, red crosses show insignificant correlations (*p* ≥ 0.05).

For unfiltered beers, strong, or very strong (according to Evans [50]) positive rank correlations were found between, e.g., antioxidant activity vs. alcohol concentration, extract content, refractive index, TPC, and Mg and Ca concentration; alcohol concentration vs. extract content, refractive index, antioxidant activity, and Mg concentration; and extract content vs. refractive index, TPC, antioxidant activity, and Mg concentration (Figure 1A). The influence of parameters such as the type of fermentation, color, the content of alcohol, and extract on the antioxidant properties of beers was also investigated by Polak et al. [30] by using two-way hierarchical clustering and an analysis of variance. They found that the antioxidant activity of beers depends significantly on the content of the extract and the color of the beer, which is in agreement with our findings. However, they stated that neither the kind of fermentation nor the alcohol content affects the antioxidant activity of beers. Moura-Nunes et al. [29] used PCA in order to discriminate beer samples according to ethanol content, bitterness, and refractive index. They also used the PLS method to correlate physicochemical attributes (density, ethanol content, bitterness, and refractive index) with antioxidant activity of beers. They found that similar to this study, TPC and antioxidant capacity of beers were correlated with ethanol concentration and refractive index. This study revealed that some of the rank correlations exist irrespectively to the data filtering by color and fermentation method (e.g., alcohol concentration vs. extract content and Mg concentrations and extract content vs. refractive index). However, some correlations exist only for specific types of beers. TPC shows a monotonic relationship with antioxidant activity and Mg for pale (Figure 1B), dark (Figure 1C), and bottom-fermented beers (Figure 1E). The correlation between TPC and Mg in dark beers was also described by Rodrigo et al. [18], who studied the influence of style and origin on the mineral content of beers from the United Kingdom. Moreover, a positive correlation was found for antioxidant activity and Mn for pale beers (Figure 1B). This is in agreement with the results obtained by Sulaiman et al. [51]. Relationships between antioxidant activity and Mg and Ca for dark (Figure 1C) and bottom-fermented beers (Figure 1E); Mg and Mn for pale (Figure 1B); and top-fermented beers (Figure 1D); and Na and Fe for bottom-fermented beers (Figure 1E) were found. Only one negative and strong correlation was reported between antioxidant activity and Na concentration in top-fermented beers (Figure 1D).

One of the objectives of this study was to evaluate the correlation between metal and total phenolic content in beers. A literature review shows that such relationships might exist for some matrices. For example, Kostic et al. [52] found a correlation between Mn, Fe, and TPC in extracts of *Origanum vulgare* L., as well as between the amount of flavonoids and Zn, Cu, and Mn in extracts of *Delphinidum consolida* L. Perna et al. [53] demonstrated a positive correlation between TPC and metals such as Fe, Co, Cr, Zn, and Pb in honey. Our studies revealed that with regard to beer, there were no correlations between TPC and investigated macro- and microelements (except for Mg, Ca, and Mn in some types of beers), which may indicate that the main phenolic compounds present in beer do not form complexes with the metals tested. The investigations performed by Pohl et al. [54] using sorption columns of different properties revealed that a majority of the Cu in beer (74–82%) was present in residual fraction. Out of the remaining Cu, 10-14% was present in the form of hydrophobic species (including phenolic-bound species), and 12–13% was present as cationic or free Cu. Mn was mainly present in cationic form, and only 0.3–8.2% was identified as organically bound [11,32]. Only Fe was to a large degree associated with organic compounds (31–56%), probably the phenolics, phytic acids, and low molecular weight organic acids. However, the stability constants of the Fe-tannic acid complex were decreased by about 8-12 orders of magnitude at pH 5.0 and lower [55]. The pH of the 29 tested beer samples was in the range of 3.91–4.54.

**Figure 2:**
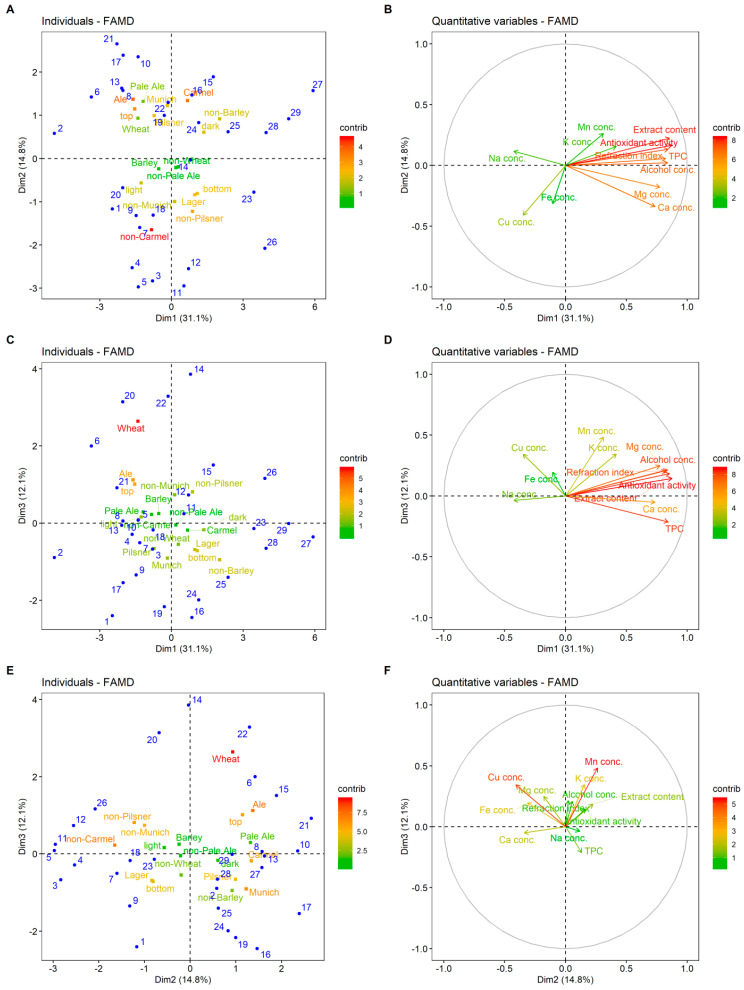
Factor maps for the first three dimensions (factors) obtained from the factor analysis of mixed data. (**A**,**C**,**E**): Individual (different beers listed in Table 2) in blue and qualitative variable categories colored according to their contribution to dimensions. (**B**,**D**,**F**): Quantitative variables colored according to their contribution to dimensions.

Factor maps for the first three dimensions (principal components) obtained from FAMD, representing individuals (different beers) and different categories (qualitative and quantitative variables), are presented in Figure 2. Individuals with negative Dim1 values were generally ale, top-fermented beers with higher than average concentration of Na (beers 2, 6, 8, 13, and 17). Positive values of this dimension indicate caramel, bottom-fermented beers with higher than average values of antioxidant activity, TPC, and Mg concentration (beers 23–25 and 27–29). Individuals with positive Dim2 values were generally described as top-fermented, ale, pilsner, Munich, and caramel style of beers (beers 6, 8, 13, 16, 19, 22, and 27; Figure 2A). For example, beers 25–29 have higher than average values of extract content, antioxidant activity, TPC, and other eigenvectors with positive Dim1, while beers 6, 8, 13, and 20 show the opposite (Figure 2A,B). Beers lying in the first and second quadrant, like beers 14, 20, and 22 of Figure 2E, generally had higher than average Mn concentration, while those in the third and fourth quadrant beers 3, 9, 16, 19, 24, and 25, had lowered Mn concentration. Similarly, beers lying in the second and third quadrant of Figure 2E had higher concentrations of Cu. These results are similar to the outcomes of Alcázar et al. [9], who stated that Mn, Mg, and K were the most important variables for beer classification purposes.

**Figure 3:**
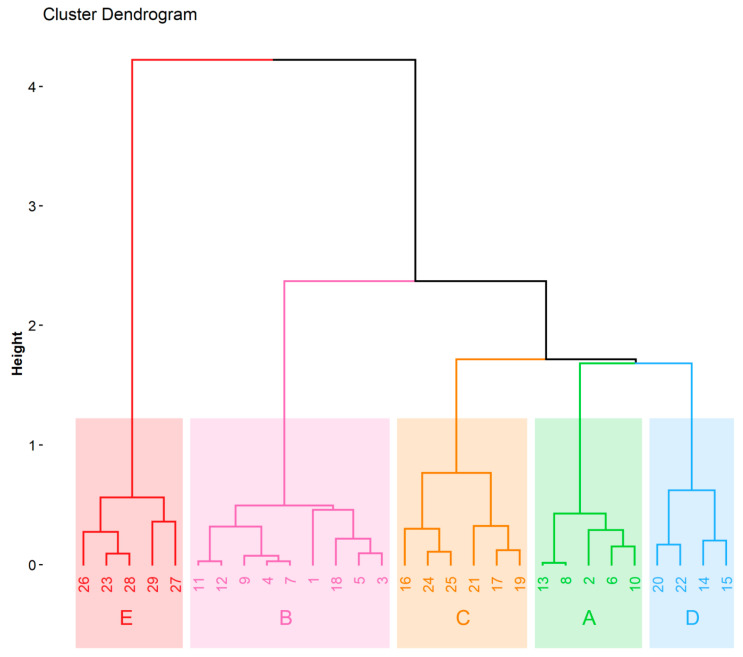
Dendrogram from hierarchical clustering on principal components, created using 6 dimensions (78.3% of explained variance) from the factor analysis of mixed data (FAMD) model and showing the clustering of beers.

Cluster analysis is the chemometric method exploited for multivariate data interpretation. The clusters (groups of similarity) can be obtained with respect to the objects of interests (which are described by various variables) or with respect to variables identifying the objects. The chemometric data interpretation in the present study was based on an input matrix consisting of 29 objects (beer samples) described by 6 dimensions of the input data matrix obtained from the FAMD model. The variables taken into account are listed in Table 5 (qualitative variables) and Table 6 (quantitative variables). The graphical output of the hierarchical clustering on principal components is a dendrogram shown in Figure 3. HCPC clustered beers into five groups—clusters A-E. All the beers assigned to the cluster A were an ale, pilsner, had a pale color, and were top-fermented. This cluster also had 60% of the pale ale beers, while the remaining 40% of beers were non-pale ale. The Ca and Mg concentration values in this cluster were lower than average. In cluster B, beers had an extract content and antioxidant activity lower than the average. This cluster contained non-caramel style and lager beers from bottom fermentation (88.88%) with a pale color (88.88%). The cluster C represents Munich, pilsner, and caramel style beers with a dark color. Cu concentration was lower than average in the C cluster. Beers assigned to the D cluster were the ale type from top fermentation, and 75% of them were wheat. They also had higher than average concentrations of Mn. The cluster E contained only dark beers with antioxidant activity, TPC, extract content, refractive index, alcohol concentration, Ca concentration, and Mg concentration higher than average. Overall, 37.93% of beers were ale, while the remaining 62.07% were lager. About 50% of the beers had a pale color. Most of the beers represented non-wheat style (82.76%) and/or non-pale ale (86.21%). Among the 29 beers tested, 13 beers were from small breweries (nine of them from the brewery located in Bialystok). Beers from the small local brewery had lower than average content of Ca, Mg (beers 2, 6, 8, 10, and 13; cluster A), and Cu (beers 17, 19, and 21; cluster C), as well as lower extract content and antioxidant activity (beer 9; cluster B; Figure 3). Beer 18 from another small brewery (cluster B) revealed lower than average extract content and antioxidant activity, while beers 15, 20, and 22 (cluster D) exhibited higher than average Mn content.

## 3. Materials and Methods

### 3.1. Samples

Polish beers analyzed in this study were purchased from the local market in 2017. They included 13 craft beers produced by local breweries, among them 9 beers produced by a brewery located in Bialystok. Beers were classified according to the Guidelines of the Beer Judge certification program [56] in two types, ale (*n* = 12) and lager (*n* = 17). Beers with fast-acting (top-fermenting) yeast, which leave behind residual sugars, are termed “ales”. Beers with slower and longer acting (bottom-fermenting) yeast, which remove most of the sugars thus leaving a clean and dry beer, are termed “lagers”. Beers were selected in order to have a representative number of beers with different color characteristics (pale (*n* = 14) and dark (*n* = 15)), different fermentation locations (top (*n* = 12) and bottom (*n* = 17)), and varying extract and alcohol content (standard strength (*n* = 13), high-strength (*n* = 14), and very-high-strength (*n* = 2)). The list of beers and their characteristics are presented in Table 2. Brand names were omitted and denoted by numbers 1–29. The levels of alcohol and extract contents are those claimed by the production company and labeled on the commercial products. Beers were stored in a refrigerator at 4 °C and analyzed immediately upon opening.

### 3.2. Instrumentation

The metals concentrations were determined using a high-resolution continuum source flame atomic absorption spectrometer ContrAA 700 (Analytik Jena AG, Jena, Germany) equipped with high-pressure xenon short-arc lamp XBO 301 (GLE, Berlin, Germany) operating in a “hot-spot” mode. High-purity acetylene (Air Liquide, Kraków, Poland) was used as fuel gas. The air-acetylene flame was used for the atomization of elements. 

Refractive index was determined using a manual refractometer RL-1 (PZO, Warsaw, Poland) at a temperature of 21 °C. Spectrophotometer UV-VIS (Hitachi U-3900H, Tokyo, Japan ) was used for absorbance measurements and spectra recording, using a quartz cuvette of 1 cm optical path length. The pH measurements were carried out with pH meter inoLab pH Level 1 (WTW, Weilheim, Germany) equipped with a glassy electrode. Ultrasound bath Sonorex Digiplus (Bandelin, Germany) was used for degassing beer samples.

### 3.3. Reagents and Materials

All reagents used in this study were of analytical grade or higher. Stock solutions for atomic absorption spectroscopy of sodium (I), potassium (I), and calcium (II) (1000 mg/L in 0.5 mol/L HNO_3_, Merck, Darmstadt, Germany), magnesium (II), iron (III), copper (II), and zinc (II) (1000 ± 4 mg/L in 2% HNO_3_, Fluka, Switzerland), and manganese (II) (1003 ± 4 mg/L in 2% HNO_3_, Fluka, Switzerland) were used for the preparation of standards. Lanthanum (III) nitrate (La(NO_3_)_3_∙6H_2_O; BDH, Poole Dorset, England) was used as a releasing agent (matrix modifier) for calcium determination. Nitric acid (69.5%, Trace Select, Fluka, France) and hydrochloric acid (37%, fuming, Trace Select, Fluka, France) were used for the preparation of standards and samples. Nitric acid and hydrogen peroxide (30%, Chempur, Poland) were used for sample digestion. All solutions were prepared daily in deionized water obtained from the Milli-Q (MQ) water purification system (Millipore, Burlington, MA, USA). Potassium persulfate and sodium carbonate were purchased from Chempur (Piekary Śląskie, Poland). Ethanol (96%), nitric acid (65%), and hydrochloric acid (35–38%) were supplied by POCH (Gliwice, Poland). Gallic acid, Trolox (6-hydroxy-2,5,7,8-tetramethylchromane-2-carboxylic acid), ABTS (diammonium 2,2′-azino-bis(3-ethylbenzothiazoline-6-sulphonate)), and Folin–Ciocalteu reagent were obtained from Sigma–Aldrich (Steinheim, Germany). A stock solution of Trolox (2 × 10^−3^ mol/L) was prepared in 6% (*v/v*) ethanol in MQ water, while a stock solution of gallic acid (1000 μg/mL) and ABTS (7 × 10^−3^ mol/L) was prepared in MQ water and kept in the dark at 4 °C. Working standards of gallic acid were prepared daily with dilution with MQ water and Trolox with 6% (*v/v*) ethanol in MQ water solution. Certified reference material of mixed Polish herbs (MPH-2) was obtained from the Institute of Nuclear Chemistry and Technology (Warsaw, Poland).

### 3.4. Procedures

#### 3.4.1. Preparation of Beer Samples for Analysis

All beer samples were degassed in an ultrasonic bath (power 190 W, 30 min). Beer samples were diluted 20-fold with 1% La in 1% HCl for determination of Ca, and diluted 10-fold with 1% HCl for determination of the total concentrations of K, Na, and Mg. Before determination of Cu, Fe, and Mn by HR CS FAAS, beer samples (15 mL portions) were digested with concentrated nitric acid (1.625 mL) and 30% hydrogen peroxide (1.5 mL) by modified EPA method 3050B [33]. The mixtures were heated on a hot plate at 100 °C in quartz crucibles covered with watch glasses until complete mineralization (approximately 1 h). After cooling, the resulting solutions were transferred to vials and diluted to 15 mL with MQ water. For each beer, three independent samples were prepared. Respective blank samples were prepared accordingly. In order to determine the total phenolic content and the total antioxidant activity, the degassed beer samples were diluted 8-fold and 5-fold with MQ water, respectively.

#### 3.4.2. Determination of Elements by HR CS FAAS

Measurements were carried out at the main atomic lines for Zn (213.857 nm), Mn (279.482 nm), Fe (248.328 nm), Cu (324.754 nm), and Ca (422.6728 nm) and at the secondary atomic lines for Na (330.237 nm), K (404.414 nm), and Mg (202.582 nm). All elements were atomized using an air-acetylene flame. The optimum acetylene and airflow rates were 45 and 470 L/h (Cu), 55 and 470 L/h (Zn), 60 and 470 L/h (Fe, Na, K, and Mg), 75 and 470 L/h (Mn), and 80 and 470 L/h (Ca). The number of pixels used for each element is listed in Table 1. All the measurements were carried out in at least triplicate. The dynamic background correction technique “with the reference” was used. 

The concentration of elements in beer samples was determined using the external calibration graph method. Multielement calibration solutions containing K (1.0–100.0 mg/L), Na (1.0–40.0 mg/L), and Mg (0.5–12.0 mg/L) in 1% HCl were used for sequential determination of macroelements in beers. Multielement calibration solutions containing Fe (0.2–2.0 mg/L), Cu (0.01–1.2 mg/L), and Mn (0.05–0.6 mg/L) in 10% HNO_3_ were used for sequential determination of microelements in digested beer samples. Standard solutions of Ca (1.0–10.0 mg/L) were prepared in 1% La (10 g/L) and 1% HCl.

The certified reference material (CRM) of mixed Polish herbs (MPH-2) was used for controlling the method trueness. The material was digested with HNO_3_ and H_2_O_2_ in Teflon vessels in a closed microwave system (Ethos plus, Milestone, Sorisole (BG), Italy). In the first step, samples (0.5 g) were placed into vessels with 6 mL HNO_3_ and left for 3 h. Next, 1 mL of 30% H_2_O_2_ was added, and the heating program recommended for the digestion of plants was run. The digests were transferred into PP vessels, diluted properly with MQ water, and analyzed using developed procedures.

#### 3.4.3. Determination of the Total Phenolic Content

The total phenolic content of beer was determined spectrophotometrically with an FC reagent [57]. A calibrating curve was plotted using gallic acid as a standard. The beer samples were diluted with MQ water to fit the concentration of phenolic compounds to the linear calibration range of gallic acid. Results were expressed as mg GAE per liter.

#### 3.4.4. Determination of the Total Antioxidant Capacity

The analysis of the total antioxidant activity of beer samples was carried out using an ABTS decolorization assay, as described by Re et al. [58] with adaptations. ABTS radical cation stock solution (ABTS^•+^) was generated by mixing an ABTS with potassium persulfate. The ability of antioxidants to scavenge the ABTS^•+^ chromophore was measured spectrophotometrically and compared to the antioxidant activity of Trolox. Two milliliters of ABTS^•+^ solution were mixed with 100 μL of beer sample or Trolox standards. The absorbance was measured exactly 3 min after mixing the solutions. Quantification was performed using a calibration curve of Trolox, and results were expressed as μmol of Trolox equivalent (TE) per liter.

### 3.5. Statistical Analysis

The R programming language was used to perform all statistical computations and analyses, as well as to prepare plots [59]. Quantitative variables from the dataset (Table 2) were assessed for normality using the Shapiro–Wilk test (“stats” package). Data were considered normally distributed for the test *p* ≥ 0.05. Since only the refractive index, TPC, Na, K, Mg, Mn, and Cu concentrations were considered Gaussian distributed for unfiltered beers (Table S3 from Supplementary Material), Spearman’s rank correlations were used to verify the monotonic relationship between each variable. Spearman’s correlations (significant for *p* < 0.05) were calculated using the “rcorr” function from the “Hmisc” package and plotted as a heatmap using the “corrplot” package [60].

In order to detect the structural and general regularities between beers and study the relationships between all the variables (both qualitative and quantitative variables), factor analysis of mixed data combined with hierarchical clustering on principal components was performed. A function “FAMD” (parameter “ncp” set to 6) and a function “HCPC” (parameter “consol” set to FALSE) from the “FactoMineR” package were used for this purpose [61]. To simplify further interpretation of the obtained FAMD model, only the first three dimensions (principal components/factors) were used (about 57.93% of explained variance), as suggested by the scree plot (Figure S1 from Supplementary Material) that was created using the “fviz_eig” function from the “factoextra” package. A plot of cos^2^ showing the quality of representation for variables on the factor map (Figure S2 from Supplementary Material) was prepared using the “corrplot” package. FAMD individual and variable factor maps were created using the “fviz_ind” and “fviz_var” functions from the “factoextra” package. Furthermore, an interactive 3D scatter plot of FAMD individuals was prepared using the “plotly” package (Figure S3 from Supplementary Material). Clustering using HCPC was performed using six dimensions from the FAMD model that together explain about 78.3% of the variance. A dendrogram (Figure 3) of obtained clusters was created using the “fviz_dend” function from the “factoextra” package [62].

## 4. Conclusions

In this paper, the fast HR CS FAAS method for sequential determination of three macro- and three microelements in beers was described for the first time. Reliable results for Na, K, and Mg concentrations were obtained when beer samples were diluted 10-fold with 1% HCl, while Ca concentration was determined from a 20-fold dilution and addition of a 1% La modifier. For sequential determination of Cu, Fe, and Mn, beer samples were digested with HNO_3_ and H_2_O_2_ at a hot place. The analytical performance of the HR CS FAAS based method with the developed sample preparation procedure was satisfactory. The precision of repeatedly prepared and measured sample solutions was very good, i.e., 0.8–8.0% (as RSD), and the limit of detection was in the range of 0.45 (Mn)–94 to µg/L (Na). Along with HR CS FAAS detection, the developed method will be a powerful tool for direct analysis of beers for the purpose of quality and safety evaluation.

Considering the daily needs of adults for macro- and microelements, beer turned out to be a rich source of Mg (8–21%) and K (5–12%). The rest of the studied elements (Na, Ca, Fe, Mn, and Cu) covered from 0.2 to 7% of the daily need. The highest values of TPC and antioxidant activity were found in dark beers, especially porters and bocks. This study proved that the mineral content, TPC, and antioxidant activity of Polish beers varied remarkably across the beer types and brands. Performed statistical analyses indicated many positive, monotonic relations between studied parameters (e.g., antioxidant activity vs. TPC, extract content, alcohol concentration, refractive index, Mg, and Ca concentration). Some of the relations were reported only for beers with specific color or fermentation methods, such as antioxidant activity vs. Mn concentration for pale beers, or TPC vs. antioxidant activity and Mg concentration for pale, dark, and bottom-fermented beers. Only one negative strong rank correlation was found between antioxidant activity and Na concentration in top-fermented beers. Hierarchical clustering on model build-up by factor analysis of mixed data not only revealed the generic structure and characteristic of the beer dataset, but it also helped to group and compare specific beers.

## Figures and Tables

**Table 1:** Instrumental parameters and analytical characteristics of methods for determination of target macro- and microelements by high-resolution continuum source flame atomic absorption spectrometry (HR CS FAAS). Sequential determination of Na, K, and Mg in beer was performed for samples diluted with 1% HCl; sequential determination of Mn, Fe, and Cu was performed in samples of beer digested with HNO_3_ + H_2_O_2_.

Element	Wavelength, nm	Number of Pixels	Slope, L/mg	Linear Calibration Range, mg/L	Working Calibration Range, mg/L	LOD ^1^, µg/L	LOQ ^2^, µg/L	Precision, % (*n* = 7)	Between-Day Precision, % (*n* = 5)	Reproducibility in Beer, % (*n* = 3)
**Sequential Determination of 8 Elements in 1% HCl**										
**Na**	330.2370	5	0.0127	1.0–40.0	1.0–66.0	12	40	0.9	5.1	1.8
**K**	404.4140	3	0.0046	10–200	10–200	34	128	0.6	8.0	1.1
**Mg**	202.5820	5	0.0729	1.0–7.0	1.0–12.0	24	80	2.3	1.4	1.1
**Ca**	422.6728	5	0.0538	1.0–8.0	1.0–35.0	3.6	12	3.2	3.8	-
**Mn**	279.4820	7	0.5611	0.05–0.6	0.05–2.5	0.26	0.88	0.6	3.7	-
**Fe**	248.3281	7	0.2029	0.05–2.0	0.05–6.1	3.5	11	0.4	2.7	-
**Cu**	324.7542	3	0.2765	0.01–1.2	0.01–2.6	16	54	2.3	5.2	-
**Zn**	213.8572	3	0.8715	0.01–0.5	0.01–1.6	1.7	5.7	2.1	4.8	-
**Sequential Determination of 3 Microelements in 10% HNO_3_**										
**Mn**	279.4820	7	0.6172	0.05–0.6	0.05–2.2	0.45	1.52	0.6	4.5	0.9
**Fe**	248.3281	7	0.2110	0.05–2.0	0.05–6.1	2.8	9.4	0.9	3.6	1.9
**Cu**	324.7542	3	0.2889	0.01–1.2	0.01–2.6	6.7	22.4	2.2	4.7	4.5
**Determination of Ca in 1% HCl + 1% La**										
**Ca**	422.6728	5	0.0365	0.5–20	-	85	285	0.9	1.3	7.3

^1^ LOD—limit of detection, ^2^ LOQ—limit of quantification.

**Table:** (**A**)

No.	Type	Type of Malt						Fermentation Method	Color
		Pilsner	Barley	Pale Ale	Munich	Wheat	Carmel		
1	Lager	+						bottom	pale
2	Ale ^1^	+	+	+	+			top	pale
3	Lager		+					bottom	pale
4	Lager		+					bottom	pale
5	Lager		+					bottom	pale
6	Ale ^1^	+	+	+		+	+	top	pale
7	Lager	+	+					bottom	pale
8	Ale ^1^	+	+		+		+	top	pale
9	Lager ^1^	+	+		+			bottom	pale
10	Ale ^1^	+	+	+	+		+	top	pale
11	Lager		+					bottom	pale
12	Lager		+					bottom	pale
13	Ale ^1^	+	+		+		+	top	pale
14	Ale		+			+		top	pale
15	Ale ^1^	+					+	top	pale
16	Lager	+			+		+	bottom	dark
17	Ale ^1^	+	+		+		+	top	dark
18	Lager ^1^		+					top	dark
19	Lager ^1^	+	+		+		+	bottom	dark
20	Ale ^1^		+			+		top	dark
21	Ale ^1^	+	+		+	+	+	top	dark
22	Ale ^1^		+			+	+	top	dark
23	Lager ^2^		+				+	bottom	dark
24	Lager ^2^	+			+		+	bottom	dark
25	Lager ^2^	+	+		+		+	bottom	dark
26	Lager ^3^		+					bottom	dark
27	Lager ^3^	+			+		+	bottom	dark
28	Lager ^3^		+		+		+	bottom	dark
29	Lager ^3^			+			+	bottom	dark

^1^ craft beers, ^2^ bock, ^3^ porter.

**Table:** (**B**)

No.	pH	Alcohol Conc. (% *v*/*v*)	Density (g/mL)	Extract (%)	Refractive Index	Total Phenolic Content (mg/L) ± SD	Antioxidant Activity (μmol TE/L) ± SD
1	4.28	4.2	1.0045	10	1.340	518 ± 5	51.6 ± 0.7
2	4.27	4.3	1.0069	10	1.340	484 ± 15	46.1 ± 3.1
3	4.09	5.2	1.0040	11.3	1.341	613 ± 8	80.5 ± 4.3
4	4.27	6	1.0062	12	1.341	540 ± 10	54.9 ± 2.4
5	4.21	6	1.0075	12.1	1.343	542 ± 12	56.2 ± 1.2
6	4.54	5.3	1.0104	12.5	1.343	529 ± 30	57.1 ± 2.3
7	4.25	6	1.0083	13.2	1.342	598 ± 2	66.8 ± 0.9
8	4.45	5.2	1.0096	13.8	1.345	630 ± 15	61.2 ± 0.5
9	4.46	5	1.0097	13.8	1.344	682 ± 37	53.2 ± 2.1
10	4.33	5.5	1.0040	14	1.342	660 ± 18	104 ± 2
11	4.25	6.5	1.0102	14.5	1.344	763 ± 17	81.2 ± 0.7
12	4.42	7.4	1.0121	14.5	1.343	786 ± 11	74.3 ± 2.2
13	4.38	6	1.0027	15	1.342	625± 21	58.9 ± 3.9
14	4.42	6	1.0177	16	1.346	896 ± 10	104 ± 1
15	4.49	8.4	1.0155	18.1	1.347	859 ± 5	89.4 ± 0.8
16	4.21	4.1	1.0213	11.9	1.343	1044 ± 36	103 ± 2
17	4.15	5	1.0071	12.8	1.341	743 ± 8	76.3 ± 1.1
18	4.19	5.2	1.0259	14.8	1.347	759 ± 12	67.7 ± 1.8
19	4.26	5.5	1.0115	12.8	1.343	816 ± 2	76.0 ± 1.6
20	4.25	5.6	1.0070	13.1	1.343	473 ± 3	72.8 ± 0.5
21	3.91	5.2	1.0065	13.5	1.340	627 ± 25	55.4 ± 0.5
22	4.19	6.4	1.0150	14.8	1.345	307 ± 16	108 ± 2
23	4.32	6.5	1.0156	15.1	1.346	1185 ± 15	146 ± 2
24	4.12	7	1.0127	16	1.345	708 ± 16	69.9 ± 2.2
25	4.53	6.5	1.0193	16	1.345	894 ± 22	108 ± 1
26	4.50	8	1.0225	18.1	1.348	927 ± 25	123 ± 1
27	4.36	9.5	1.0121	21	1.348	1266 ± 24	142 ± 2
28	4.15	8.9	1.0231	22	1.351	974 ± 5	106 ± 1
29	4.14	9.2	1.0140	22	1.349	1108 ± 28	145 ± 3
**Overall mean**	4.29	6.19	1.0118	14.64	1.344	743	84.1
**Min**	3.91	4.10	1.0040	10.00	1.340	307	46.1
**Q_1_**	4.19	5.20	1.0070	12.80	1.342	598	55.9
**Q_2_**	4.27	6.00	1.0104	14.00	1.343	708	76.0
**Q_3_**	4.42	6.50	1.0155	16.00	1.346	894	104
**Max**	4.54	9.50	1.0259	22.00	1.351	1266	146

Q_1_—lower quartile; Q_2_—median; Q_3_—upper quartile.

**Table:** (**C**)

No.	Metal conc. (mg/L) ± SD				Metal conc. (μg/L) ± SD		
	Na	K	Mg	Ca	Fe	Mn	Cu
1	21.7 ± 0.2	469 ± 4	80.2 ± 1.2	41.3 ± 2.3	69.5 ± 0.6	108 ± 0	30.1 ± 1.8
2	46.8 ± 0.1	481 ± 3	64.0 ± 1.0	31.5 ± 0.6	220 ± 4	67.3 ± 0.3	70.5 ± 4.8
3	16.6 ± 0.3	410 ± 3	106 ± 1	103 ± 0	63.6 ± 5.2	144 ± 0	55.1 ± 3.0
4	74.2 ± 1.3	595 ± 2	108 ± 1	67.8 ± 3.9	159 ± 2	122 ± 2	63.2 ± 1.8
5	25.8 ± 0.1	515 ± 6	97.7 ± 0.3	45.8 ± 0.8	1872 ± 2	146 ± 1	33.3 ± 0.1
6	44.5 ± 0.5	541 ± 4	87.1 ± 1.0	30.3 ± 0.7	150 ± 0	145 ± 1	84.1 ± 2.9
7	70.3 ± 1.6	533 ± 2	131 ± 2	41.9 ± 1.3	419 ± 3	159 ± 3	53.8 ± 3.6
8	74.0 ± 2.0	685 ± 8	99.6 ± 1.0	47.8 ± 1.1	193 ± 1	127 ± 2	65.6 ± 0.8
9	30.4 ± 1.0	585 ± 3	122 ± 3	34.5 ± 1.0	159 ± 1	76.2 ± 0.7	85.8 ± 3.1
10	32.3 ± 0.3	609 ± 2	110 ± 1	38.9 ± 3.8	301 ± 3	155 ± 0	36.1 ± 2.3
11	33.6 ± 0.2	515 ± 4	122 ± 2	108.0 ± 6	365 ± 6	148 ± 2	76.7 ± 4.5
12	21.3 ± 0.3	554 ± 1	158 ± 2	53.0 ± 1	153 ± 3	181 ± 3	75.9 ± 2.7
13	49.4 ± 0.8	640 ± 5	99.5 ± 0.2	32.6 ± 2.4	135 ± 1	125 ± 1	63.3 ± 1.5
14	20.3 ± 0.8	792 ± 11	141 ± 1	58.2 ± 1.5	157 ± 5	235 ± 2	53.3 ± 2.3
15	46.4 ± 1.1	815 ± 1	131 ± 1	56.0 ± 1.9	397 ± 2	225 ± 2	38.4 ± 1.4
16	22.5 ± 0.4	367 ± 10	106 ± 2	63.1 ± 0.3	252 ± 2	277 ± 1	21.0 ± 0.7
17	43.5 ± 0.6	536 ± 5	84.3 ± 2.7	27.3 ± 0.3	135 ± 1	65.4 ± 0.2	13.3 ± 1.1
18	61.0 ± 1.4	427 ± 6	95.8 ± 0.1	54.2 ± 1	508 ± 6	81.4 ± 0.5	43.5 ± 1.9
19	54.2 ± 1.1	520 ± 13	112.4 ± 0.9	42.6 ± 0.4	238 ± 10	153 ± 1	20.0 ± 0.3
20	7.75 ± 0.04	528 ± 6	102 ± 2	19.1 ± 1.6	1199 ± 13	181 ± 1	68.5 ± 0.3
21	63.6 ± 2.3	428 ± 10	108 ± 0	43.3 ± 4.6	272 ± 6	255 ± 1	33.3 ± 2.2
22	32.8 ± 0.2	512 ± 4	120 ± 0	56.4 ± 3.5	62.4 ± 1.7	277 ± 1	40.8 ± 0.5
23	31.0 ± 1.1	624 ± 2	127 ± 1	117.2 ± 4.1	362 ± 0	151 ± 1	37.3 ± 1.7
24	10.9 ± 0.1	491 ± 3	123 ± 4	50.3 ± 3.5	134 ± 2	126 ± 2	53.0 ± 2.7
25	15.0 ± 0.3	689 ± 17	140 ± 1	93.1 ± 3.4	116 ± 5	114 ± 2	33.7 ± 5.7
26	34.0 ± 1.2	742 ± 5	169 ± 0	114 ± 0	220 ± 17	144 ± 6	66.9 ± 9.2
27	17.6 ± 0.1	855 ± 16	155 ± 1	103 ± 2	286 ± 13	207 ± 10	41.3 ± 0.7
28	32.9 ± 0.5	519 ± 17	113 ± 1	84.3 ± 1	303 ± 3	141 ± 1	12.1 ± 0.1
29	17.5 ± 0.4	491 ± 3	134 ± 1	66.5 ± 0.1	140 ± 0	174 ± 0	21.7 ± 0.6
**Overall mean**	36.3	568	115	59.4	312	156	48.0
**Min**	7.75	367	64.0	19.1	62.4	65.4	12.1
**Q_1_**	21.3	491	99.6	41.3	140	125	33.3
**Q_2_**	32.8	533	112	53.0	220	146	43.5
**Q_3_**	46.8	624	131	67.8	303	181	65.6
**Max**	74.2	855	169	117	1872	277	85.8

Q_1_—lower quartile; Q_2_—median; Q_3_—upper quartile.

**Table 3:** Concentration of elements (mg/L) in beers manufactured in different countries; ranges, mean (m), and median values (M) in brackets.

Country of Origin	Na	K	Mg	Ca	Fe	Mn	Cu	Reference
Poland (*n* = 30)					0.045–0.530 (0.130) ^M^	0.053–0.47 (0.160) ^M^	0.029–0.150 (0.060) ^M^	[16]
Brazil (*n* = 4)						0.11–0.348	0.038–0.155	[12]
Poland (*n* = 18)		172–518 (309)^m^	92–220 (132)^m^		0.05–0.45 (0.14) ^m^	0.03–0.15 (0.12) ^m^	0.01–0.09 (0.04) ^m^	[35]
Poland (*n* = 6)					0.208–0.345	0.070–0.165	0.072–0.114	[11]
Germany (*n* = 15)	30.4–77.7 (46.3)^M^	442.8–570.3 (493.8) ^M^	66.8–126.7 (105.6) ^M^	45.9–95.8 (61.6) ^M^	0.07–1.41 (0.42) ^M^	0.05–0.26 (0.18) ^M^		[28]
Portugal (*n* = 18)	8.4–129.6 (24.1) ^M^	255.2–443.1 (354) ^M^	58.3–113.8 (88.8) ^M^	28–93 (54.8) ^M^	0.05–0.88 (0.24) ^M^	0.06–0.19 (0.13) ^M^		
Spain (*n* = 35)		251–563.5 (425.3) ^M^	43.1–210.4 (94) ^M^	21.8–108.5 (48.2) ^M^	0.03–0.3 (0.16) ^M^	0.03–0.35 (0.14) ^M^		
Distributed in Romania (*n*=20)		29.8–197.0	22.5–84.7	11.2–62.2	0.2–4.2	0.0042–0.2317	0.026–0.073	[17]
Britain Spain Germany	21.90–230 3.95–103 1.19–120	135–1100 22.9–496 22.9–496	60–200 42.0–110 23.7–266	40–140 9.0–86.2 3.80–108				[17]
Portugal (*n* = 4)		19–191		38–52				[36]
Thailand (*n* = 8)		109–125		43–121				
Italy (*n* = 4)		143–145		62–105				
Vietnam (*n* = 6)	17.4–81.9 (46.8) ^M^	112–135		90–204				
Distributed in UK (*n* = 125)	19.1–53.2 (41)^m^	239.8–626.2 (451) ^m^	57.3–99.8 (78) ^m^	24.1–61.5 (52) ^m^	0.198–4.073	0.09–0.35	0.003–0.633	[18]
Germany (*n* = 13)	(19.1) ^m^	(450.2) ^m^	(79.5) ^m^	(41.7) ^m^	(0.579) ^m^	(0.13) ^m^	(0.400) ^m^	
Belgium (*n* = 19)	(49.7) ^m^	(504.2) ^m^	(83.3) ^m^	(54.8) ^m^	(4.073) ^m^	(0.35) ^m^	(0.633) ^m^	
UK (*n* = 53)	(48.3) ^m^	(436.5) ^m^	(73.6) ^m^	(61.5) ^m^	(0.554) ^m^	(0.14) ^m^	(0.003) ^m^	
USA (*n* = 14)	(26.8) ^m^	(626.2) ^m^	(99.8) ^m^	(38.7) ^m^	(0.489) ^m^	(0.25) ^m^	(0.006) ^m^	

*n*—number of beer samples; ^m^ mean; ^M^ median.

**Table 4:** Hypothesis testing for the content of total phenolic compounds (TPC) and antioxidant activity.

**Question:**	**Does a Parameter in X Type of Beers Follow Normal Distribution?**					
**Statistical Test Name:**	**Shapiro–Wilk Test**					
**Parameter**	**X**		**W Statistics**	***p*** **-Value**		**Answer**
TPC	Light color (*n* = 15)		0.92480	0.2279		Yes
TPC	Dark color (*n* = 14)		0.98313	0.9890		Yes
TPC	Top fermentation (*n* = 12)		0.97288	0.9385		Yes
TPC	Bottom fermentation (*n* = 17)		0.94575	0.3928		Yes
Antioxidant activity	Light color (*n* = 15)		0.89193	0.0717		Yes
Antioxidant activity	Dark color (*n* = 14)		0.91033	0.1593		Yes
Antioxidant activity	Top fermentation (*n* = 12)		0.90478	0.1828		Yes
Antioxidant activity	Bottom fermentation (*n* = 17)		0.89343	0.0528		Yes
**Question:**	**Does a Parameter in X and Y Types of Beers Have the Same Variance?**					
**Statistical Test Name:**	**F Test**					
**Parameter**	**X**	**Y**	**F Statistics**	***p*** **-Value**		**Answer**
TPC	Light color	Dark color	0.22285	0.0087		No
TPC	Top fermentation	Bottom fermentation	0.51929	0.2736		Yes
Antioxidant activity	Light color	Dark color	0.36796	0.0744		Yes
Antioxidant activity	Top fermentation	Bottom fermentation	0.41894	0.1479		Yes
**Question:**	**Does a Parameter in X and Y Types of Beers Follow the Same Distribution?**					
**Statistical Test Name:**	**Kolmogorov–Smirnov Two-Sample Test**					
**Parameter**	**X**	**Y**	**D Statistics**	***p*** **-Value**		**Answer**
TPC	Light color	Dark color	0.51905	0.0263		No
TPC	Top fermentation	Bottom fermentation	0.42157	0.1195		Yes
Antioxidant activity	Light color	Dark color	0.52857	0.0349		No
Antioxidant activity	Top fermentation	Bottom fermentation	0.26961	0.6860		Yes
**Question:**	**Does a Parameter in X and Y Types of Beers Have Equal/Lower Means?**					
**Statistical Test Name:**	**Welch Two-Sample *t*-Test**					
**Parameter**	**X**	**Y**	**t Statistics**	***p*** **-Value ^1^**	***p*** **-Value ^2^**	**Answer**
TPC	Light color	Dark color	−2.4920	0.0225	0.0113	Lower
TPC	Top fermentation	Bottom fermentation	−2.5150	0.0182	0.0091	Lower
Antioxidant activity	Light color	Dark color	−3.1999	0.0043	0.0021	Lower
Antioxidant activity	Top fermentation	Bottom fermentation	−1.5201	0.1402	0.0701	Equal

^1^ H_0_—equal means, H_1_—unequal means; ^2^ H_0_—equal or greater means, H_1_—lower means.

**Table 5:** Results for the hierarchical clustering on principal components. Description of each cluster by the categories. Only categories that characterize each cluster are shown (*p*-value < 0.05). Values of the v-test are sorted from strongest to weakest significance in the construction of the given cluster, i.e., the higher the positive or the lower the negative value is the better/stronger description of the cluster it has. Cla/Mod indicates a ratio of beers with a specific category (modality) found in a cluster (class) to all beers with this category in the dataset (%). Mod/Cla indicates a ratio of beers having a specific category to all beers in a cluster. Global shows the percentage of beers having a specific category in the dataset.

Cluster	Category	Cla/Mod	Mod/Cla	Global	v-Test	*p*-Value
A	Type = Ale	45.455	100	37.931	2.887	0.004
A	Fermentation method = top	41.667	100	41.379	2.713	0.007
A	Style Pale Ale = Pale Ale	75	60	13.793	2.558	0.011
A	Color = light	33.333	100	51.724	2.237	0.025
A	Style Pilsner = Pilsner	31.25	100	55.172	2.088	0.037
A	Style Pilsner = non-Pilsner	0	0	44.828	−2.088	0.037
A	Color = dark	0	0	48.276	−2.237	0.025
A	Style Pale Ale = non-Pale Ale	8	40	86.207	−2.558	0.011
A	Fermentation method = bottom	0	0	58.621	−2.713	0.007
A	Type = Lager	0	0	62.069	−2.887	0.004
B	Style Carmel = non-Carmel	69.231	100	44.828	3.972	0.00007
B	Type = Lager	50	100	62.069	2.817	0.005
B	Color = light	53.333	88.889	51.724	2.576	0.010
B	Style.Munich = non-Munich	50	88.889	55.172	2.346	0.019
B	Fermentation method = bottom	47.059	88.889	58.621	2.120	0.034
B	Fermentation method = top	8.333	11.111	41.379	−2.120	0.034
B	Style.Munich = Munich	7.692	11.111	44.828	−2.346	0.019
B	Color = dark	7.143	11.111	48.276	−2.576	0.010
B	Type = Ale	0	0	37.931	−2.817	0.005
B	Style Carmel = Carmel	0	0	55.172	−3.972	0.00007
C	Style Munich = Munich	46.154	100	44.828	2.910	0.004
C	Color = dark	42.857	100	48.276	2.731	0.006
C	Style Carmel = Carmel	37.5	100	55.172	2.390	0.017
C	Style Pilsner = Pilsner	37.5	100	55.172	2.390	0.017
C	Style Carmel = non-Carmel	0	0	44.828	−2.390	0.017
C	Style Pilsner = non-Pilsner	0	0	44.828	−2.390	0.017
C	Color = light	0	0	51.724	−2.731	0.006
C	Style Munich = non-Munich	0	0	55.172	−2.910	0.004
D	Style Wheat = Wheat	60	75	17.241	2.558	0.011
D	Type = Ale	36.364	100	37.931	2.460	0.014
D	Fermentation method = top	33.333	100	41.379	2.311	0.021
D	Fermentation method = bottom	0	0	58.621	−2.311	0.021
D	Type = Lager	0	0	62.069	−2.460	0.014
D	Style Wheat = non-Wheat	4.167	25	82.759	−2.558	0.011
E	Color = dark	35.714	100	48.276	2.390	0.017
E	Color = light	0	0	51.724	−2.390	0.017

**Table 6:** Results for the hierarchical clustering on principal components. Description of each cluster by quantitative variables. Only variables that characterize each cluster are shown (*p*-value < 0.05). Values of the v-test are sorted from strongest to weakest significance in the construction of the given cluster, i.e., the higher the positive or the lower the negative value is the better/stronger description of the cluster it has.

Cluster	Variable	Mean in Category	Overall Mean	v-Test	*p*-Value
A	Ca concentration	36.214 ± 6.5	59.427 ± 27.518	−2.037	0.042
A	Mg concentration	92.096 ± 15.817	115.469 ± 23.464	−2.406	0.016
B	Extract content	12.911 ± 1.565	14.645 ± 3.067	−2.007	0.045
B	Antioxidant activity	65.155 ± 11.044	84.08 ± 28.854	−2.329	0.020
C	Cu concentration	29.05 ± 12.957	47.979 ± 20.882	−2.450	0.014
D	Mn concentration	229.62 ± 34.033	155.566 ± 56.070	2.795	0.005
E	Antioxidant activity	132.40 ± 15.628	84.08 ± 28.854	4.044	0.00005
E	Extract content	19.64 ± 2.682	14.645 ± 3.067	3.934	0.00008
E	Refraction index	1.348 ± 0.002	1.344 ± 0.003	3.794	0.00014
E	Alcohol concentration	8.42 ± 1.083	6.193 ± 1.425	3.774	0.00016
E	TPC	1091.684 ± 126.617	743.255 ± 223.154	3.771	0.00016
E	Ca concentration	96.81 ± 19.001	59.427 ± 27.518	3.281	0.001
E	Mg concentration	139.746 ± 19.784	115.469 ± 23.464	2.499	0.012

## Supplementary Materials

The following are available online. Table S1: Analytical characteristic of determination of Mg at 202.5820 nm by CS-HR-FAAS using a different number of pixels, Table S2: Results (mean ± standard deviation) for K, Mg, Ca, Fe, Mn, and Cu in CRM (MPH-2) determined by the proposed method, Table S3: Shapiro–Wilk normality test W statistics and *p*-values, Figure S1: Scree plot (a plot of the eigenvalues by the number of the dimensions/principal components) from the factor analysis of mixed data (FAMD), Figure S2: Values of cos^2^ for active variables in FAMD, showing the quality of representation for variables on the factor map (Dim1-6), Figure S3: 3D scatter plot of FAMD individuals grouped by HCPC clusters.
