# Supplementary material for: The Mineral Profile of Polish Beers by Fast Sequential Multielement HR CS FAAS Analysis and Its Correlation with Total Phenolic Content and Antioxidant Activity by Chemometric Methods
Source: Molecules. 2020 Jul 27;25(15):3402. doi: 10.3390/molecules25153402 (PMC7436273; doi:10.3390/molecules25153402)
Supplement: Supplementary file 1 [file molecules-25-03402-s001.pdf]

Supplementary materials

The mineral profile, the total phenolic content and antioxidant activity in Polish beers

E. Zambrzycka-Szelewa[a], E. Nalewajko-Sieliwoniuk[a,*], M. Zaremba[a], A. Bajguz[b] and B. Godlewska-Żyłkiewicz[a]

[a] Faculty of Chemistry, University of Bialystok, Ciołkowskiego 1K, 15-245 Białystok, Poland
[b] Faculty of Biology, University of Bialystok, Ciołkowskiego 1J, 15-245 Białystok, Poland
* Correspondence: corresponding author

Table 1. S. Analytical characteristic of determination of Mg at 202.5820 nm by CS-HR-FAAS using a different number of pixels.

Number of pixels	Slope of cabration graph, L/g	Linear range of calibration graph, mg/L	LOD, µg/L	LOQ, µg/L	Precision, %
**1**	0.0155	1 - 12	114	380	4.8
**2***	0.0286	1 - 12	77	190	2.4
**3**	0.0448	1 - 10	58	257	2.3
**5**	0.0708	0.5 - 7	60	220	2.8
**7**	0.0839	0.5 - 7	74	248	3.4
**9**	0.0894	0.5 - 7	92	307	3.5
**11**	0.0913	0.5 - 7	94	315	3.2

* based on subtraction of absorbance signal registered for 3 pixels (CP ± 1) and 1 pixel (CP).

LOD – limit of detection, LOQ – limit of quantification.

Table 2. S. Results (mean ± standard deviation) for K, Mg, Ca, Fe, Mn, and Cu in CRM (MPH-2) determined by the proposed method.

	**Content of elements in MPH-2**					
	**K, mg/g**	**Mg, mg/g**	**Ca, mg/g**	**Fe, µg/g**	**Mn, µg/g**	**Cu, µg/g**
Found value	19.22 ± 2.30	2.84 ± 0.08	10.78 ± 0.23	477 ± 5	175 ± 12	7.98 ± 0.33
Certified value	19.10 ± 1.20	2.92 ± 0.18	10.80 ± 0.70	460	192 ± 12	7.77 ± 0.53
Recovery, %	100.6	97.4	99.8	103.7	91.6	102.7

Table 3. S. Shapiro-Wilk normality test W statistics and *p*-values.

Parameter	Unfiltered		Color = light		Color = dark	
	W statistics	*p*-value	W statistics	*p*-value	W statistics	*p*-value
Alcohol conc.	0.91401	0.0215	0.92273	0.2120	0.92550	0.2636
Extract content	0.90521	0.0131	0.96937	0.8486	0.86827	0.0396
Refractive index	0.95430	0.2363	0.95311	0.5748	0.97880	0.9672
TPC	0.96947	0.5456	0.92479	0.2279	0.98313	0.9890
Antioxidant activity	0.90202	0.0109	0.89192	0.0717	0.91033	0.1593
Na conc.	0.93507	0.0745	0.8893s0	0.0655	0.92598	0.2678
K conc.	0.93400	0.0698	0.93521	0.3259	0.90703	0.1426
Mg conc.	0.98835	0.9822	0.99029	0.9995	0.97081	0.8873
Ca conc.	0.89980	0.0097	0.80579	0.0043	0.95046	0.5679
Fe conc.	0.56287	0.000000037	0.50578	0.0000039	0.67146	0.0001
Mn conc.	0.93659	0.0817	0.94217	0.4104	0.93854	0.4000
Cu conc.	0.96290	0.3868	0.94171	0.4044	0.93544	0.3630

Parameter	Fermentation method = top		Fermentation method = bottom	
	W statistics	*p*-value	W statistics	*p*-value
Alcohol conc.	0.82831	0.0200	0.02002	0.4601
Extract content	0.96625	0.8679	0.86795	0.0637
Refractive index	0.92849	0.3644	0.36443	0.3957
TPC	0.97288	0.9385	0.93852	0.3928
Antioxidant activity	0.90478	0.1828	0.18280	0.0528
Na conc.	0.97252	0.9353	0.93539	0.0039
K conc.	0.91749	0.2658	0.26580	0.2101
Mg conc.	0.98112	0.9877	0.98771	0.9251
Ca conc.	0.92551	0.3349	0.33498	0.0623
Fe conc.	0.68906	0.0006	0.00066	0.000001
Mn conc.	0.93572	0.4447	0.44474	0.0518
Cu conc.	0.96667	0.8730	0.87305	0.5037

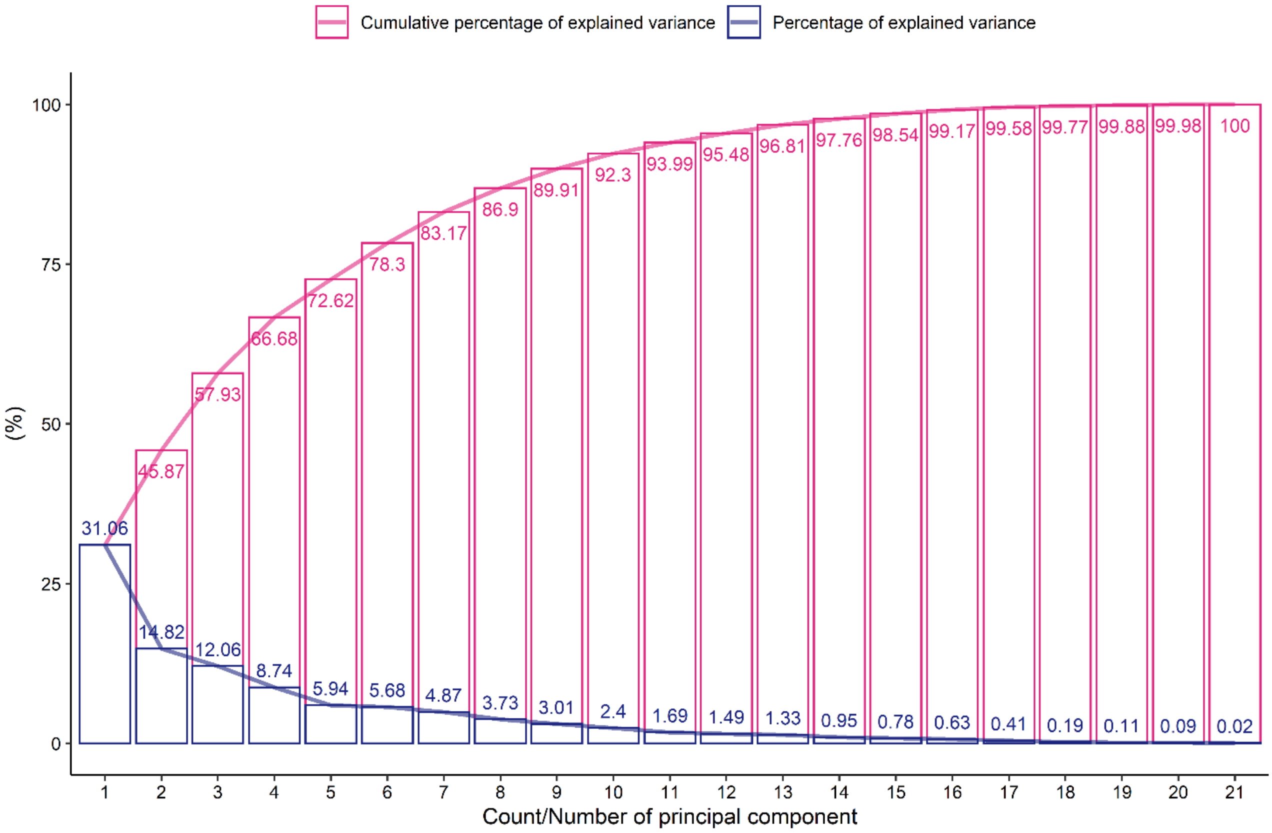

Figure 1S. Scree plot (a plot of the eigenvalues by the number of the dimensions/principal components) from Factor Analysis of Mixed Data (FAMD).

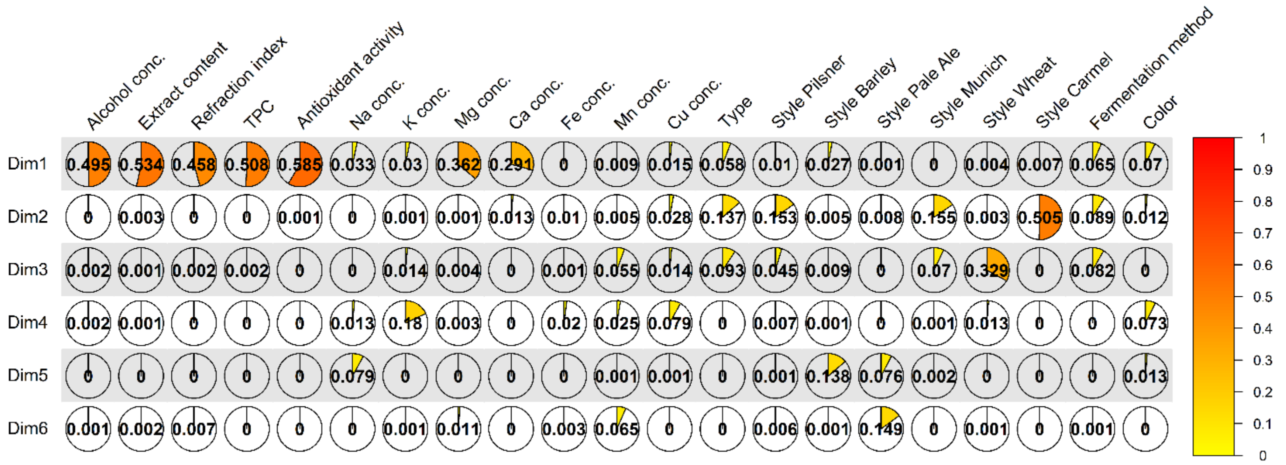

Figure 2S. Values of $\cos^2$ for active variables in FAMD, showing the quality of representation for variables on the factor map (Dim1-6).
